# Care recipients’ physical frailty is independently associated with subjective burden in informal caregivers in the community setting: a cross-sectional study

**DOI:** 10.1186/s12877-016-0355-6

**Published:** 2016-11-17

**Authors:** Thom J. Ringer, Afeez Abiola Hazzan, Courtney C. Kennedy, Sarah Karampatos, Christopher Patterson, Sharon Marr, Brian Misiaszek, Tricia Woo, George Ioannidis, Alexandra Papaioannou

**Affiliations:** GERAS Centre, Hamilton Health Sciences – St Peter’s Hospital Site, 88 Maplewood Avenue, Hamilton, ON L8M 1W9 Canada

**Keywords:** Frailty, Physical frailty, Informal caregivers, Caregiver burden, Fried Frail Scale, Zarit Burden Interview, Katz index, Activities of daily living, Functional independence

## Abstract

**Background:**

Physical frailty is associated with significant morbidity and mortality in community-dwelling older adults. Burden in informal caregivers of older adults causes significant physical and psychological distress. However, the relationship between these two clinical phenomena has not been extensively studied. This cross-sectional study evaluated the relationship between physical frailty of community-dwelling older adults attending an outpatient geriatric clinic and the subjective burden reported by their informal caregivers.

**Methods:**

We measured the following characteristics of 45 patient-caregiver dyads attending an outpatient geriatric assessment clinic: Physical frailty using the Fried Frail Scale (FFS); self-reported independence in activities of daily living (ADL) using the Katz Index; clinical diagnosis of dementia; and subjective caregiver burden using the short 12-item version of the Zarit Burden Interview (ZBI). Multivariable linear regression was performed with FFS, Katz Index score, gender, age, and diagnosis of dementia as independent variables, and ZBI score as the dependent variable.

**Results:**

Only physical frailty significantly predicted caregiver burden (β = 8.98 95% confidence interval [CI]: 2.15, 15.82).

**Conclusions:**

Physical frailty is independently associated with caregiver burden in a population of community-dwelling older adults. Despite limitations related to sample size and lack of data about caregiver characteristics, this study suggests that the relationship between physical frailty and caregiver burden merits further study.

## Background

It is well-established that providing unpaid care for an ill friend or relative over an extended period of time can impose considerable physical and emotional strain [1]. It has been observed that caregiving has all the features of a chronic stress experience, and is used as a model for studying the health effects of chronic stress [2]. Informal (i.e., unpaid) caregivers of older adults with a range of medical conditions and functional impairments experience significant physical, financial, and psychosocial hardship, and are at increased risk for psychiatric and medical morbidity [1, 3, 4].

In the geriatrics literature, caregiver burden has been defined as a multidimensional response to the negative appraisal and perceived stress resulting from taking care of an ill individual [5]. A wide range of instruments exist to measure this response, the most widely used of which is the Zarit Burden Interview (ZBI), a tool based on self-report [6–8]. The concept of informal caregiver burden has been extensively studied in the settings of dementia, cancer, and stroke [9–11].

Frailty, another prominent concept in the geriatric literature, has been defined as a medical syndrome with multiple causes and contributors that is characterized by diminished strength, endurance, and physiologic function that increases vulnerability for developing increased dependency and/or death [12]. Consensus on a definition of frailty remains elusive [13]. Fried and colleagues posit a physical frailty phenotype currently in wide use in research involving five criteria: weight loss, exhaustion, low physical activity, slowness, and weakness [14]. A recent systematic review suggests the syndrome is prevalent among older adults [14]. Physical frailty is significant because it strongly predicts adverse medical outcomes. One study found that physical frailty conferred a mortality hazard ratio of 3.09, while another concluded that it conferred an odds ratio of 3.3 for disability or mortality [15, 16]. It is also associated with length of stay and postoperative complications in older patients undergoing surgery [17].

Research to develop interventions targeting frailty is nascent [18, 19]. Key outcomes of interest have been quality of life, functional status (e.g., ability to perform activities of daily living), depression, and physical function. However, the impact of frailty-reducing interventions on caregiver burden, despite the latter’s salience and prevalence, remains relatively unexplored. Indeed, the relationship between frailty and caregiver burden as a whole has received relatively little attention [20].

Given the prevalence and health implications of frailty on the one hand and the physical and psychological burden of informal caregiving on the other, research exploring the relationship between these two phenomena is needed. The objective of this cross-sectional, real world study was to evaluate the relationship between community-dwelling older patients’ physical frailty and subjective burden in their informal caregivers.

## Methods

### Setting & participants

Our study population was a convenience sample of 45 dyads consisting of community-dwelling older adults attending a geriatric outpatient clinic in Hamilton, Ontario, Canada and their informal caregivers. Reasons for referral by the patients’ primary care physicians included cognitive impairment, falls, polypharmacy, multimorbidity, and functional decline. All patients attending the clinic received a comprehensive geriatric assessment performed by an interdisciplinary team including a registered practical nurse, case manager, and geriatrician.

All patients attending the clinic between June-December 2013 and July-August 2014 were invited to participate if they were:

1. Deemed fit to participate by a health care professional on assessment at the clinic;
2. Able to follow instructions; and,
3. Accompanied to the clinic by an informal caregiver.

The patient or their legal representative provided informed consent to participate in the study.

### Recruitment & inclusion

Of the 191 patients attending the clinic, 120 eligible patient-caregiver dyads consented. A further 75 dyads were excluded because the patient resided in a retirement home, and/or a frailty assessment and/or caregiver burden assessment was not completed. The final study sample consisted of 45 patient-caregiver dyads.

### Comprehensive geriatric assessment

Patient demographic and clinical information recorded in the course of the comprehensive geriatric assessment was abstracted (Table 1). This information included the geriatrician’s cognitive assessment, including diagnosis of dementia (e.g., Alzheimer’s, other, or mixed).

**Table 1:** Descriptive characteristics of frail and non-frail subjects

	Non-frail (FFS < 3)	Frail (FFS ≥ 3)	Sig. (2-tailed)
N	27	18	
Female gender: n (%)	9 (33.3)	13 (72.2)	0.010
Mean age: (SD)	80.5 (5.6)	82.9 (4.3)	0.122
Mean caregiver ZBI score: (SD)	13.9 (8.6)	20.7 (11.4)	0.028
Mean Katz Index (ADL) score: (SD)	10.7 (3.1)	11.1 (1.3)	0.606
Marital status: n (%)			
Single	0 (0.0)	1 (5.6)	0.225
Married	17 (63.0)	6 (33.3)	0.053
Widowed	3 (11.1)	10 (55.6)	0.001
Divorced	3 (11.1)	1 (5.6)	0.532
Incomplete	4 (14.8)	0 (0.0)	0.091
Living arrangement: n (%)			
Alone	8 (29.6)	5 (27.8)	0.896
With family	18 (66.7)	10 (55.6)	0.463
Other	0 (0.0)	1 (5.6)	0.225
Incomplete	1 (3.7)	2 (11.1)	0.340
Clinical Diagnosis of Dementia	14 (51.9)	6 (33.3)	0.230

### Caregiver burden assessment

To assess subjective caregiver burden, the accompanying informal caregiver completed the validated short (12-item) version of the Zarit Burden Interview (ZBI) [21]. The ZBI yields a score from 0 to 48, with higher scores indicating a greater degree of burden.

### Frailty assessment

Research assistants assessed patients’ physical frailty using the widely used Fried Frail Scale (FFS) criteria: reported weight loss of >10 lbs in the past year; reported level of exhaustion over the last week; reported physical activity in the past year expressed as average kcal/week; measured time to walk 4.57 m; and dominant hand grip strength in kg using a hand-held dynamometer [22, 23]. Patients meeting 3 or more of the FFS criteria (i.e., FFS ≥ 3) were designated as frail. The frail criteria were used as described in the original study by Fried and colleagues [23].

### Functional assessment

Each patient’s self- or caregiver-reported ability to perform each of the 6 activities of daily living of the Katz Index of ADLs was recorded [24]. These activities consist of bathing, dressing, toileting, transferring, continence, and feeding. For each activity, a score out of 2 was assigned (2 = needs no help; 1 = needs some help; 0 = unable to do at all), yielding a total score out of 12. Lower scores indicated decreased level of independence in activities of daily living.

### Analysis

Univariate and multivariable linear regressions were performed to determine the relationship between physical frailty (FFS ≥ 3 vs. FFS < 3) of community-dwelling older patients attending an outpatient geriatric clinic and the subjective burden reported by their informal caregivers as measured by ZBI score. Included covariates consisted of age (years), gender, Katz Index of ADLs, and diagnosis of dementia (yes/no).

## Results

### Descriptive characteristics

Of the 45 patients, 18 (40%) were frail (FFS ≥ 3). Descriptive characteristics of frail and non-frail patients are displayed in Table 1.

### Analysis

Caregiver ZBI scores were 20.7 (11.4) and 13.9 (8.6) for those providing care to frail patients compared with non frail patients, respectively (Table 1).

In univariate analysis, frailty was significantly associated with caregiver burden (β = 6.80, 95% confidence interval [CI] 0.77, 12.83) (Table 2). Age, gender, clinical diagnosis of dementia, and Katz Index score were not significantly associated with caregiver burden (Table 2).

**Table 2:** The relationship between a patient’s characteristics and caregiver burden

	Multivariate Analysis			Univariate Analysis		
		95 % CI			95 % CI	
	β coefficient	Upper	Lower	β coefficient	Upper	Lower
Frail (FFS ≥ 3)	8.98	2.15	15.82	6.80	0.77	12.83
Age (years)	−0.09	−0.71	0.53	0.04	−0.56	0.65
Gender (female)	−4.86	−11.55	1.83	−1.97	−8.20	4.25
Clinical diagnosis of dementia (yes)	−1.35	−7.72	5.03	−2.15	−8.41	4.11
Katz Index (ADL) score	−0.27	−2.94	2.39	−0.30	−3.00	2.40

In multivariable analysis, frailty was significantly associated with caregiver burden (β = 8.979, 95% CI 2.233, 15.725) (Table 2). Age, gender, clinical diagnosis of dementia, and Katz Index score were not significantly associated with caregiver burden (Table 2).

## Discussion

Our cross-sectional study of 45 caregiver-recipient dyads attending an outpatient geriatric clinic found that self-reported subjective caregiver burden was higher in those caring for frail patients. Our study also found that caregivers of patients who were diagnosed with dementia did not experience significantly more burden than caregivers of patients without dementia. In contrast to previous studies, we found that a patient’s level of independence in ADLs was not significantly associated with increased caregiver burden [5].

### Strengths & Limitations

Our study is one of the first to directly explore the relationship between physical frailty and caregiver burden using widely used and validated tools to measure each (the FFS and ZBI respectively). Its real world setting – that of a specialized geriatric assessment clinic employing an interdisciplinary care model – also reflects its potential relevance to clinical practice.

Our study findings should be viewed in the context of its design. Our sample size was modest and a larger sample size would increase the statistical power to detect additional factors that may be related to caregiver burden. In addition, our cross-sectional analysis means that causal relationships are difficult to interpret. Reasons for non-completion of the caregiver burden interview or FFS were not recorded, so selection effect could not be evaluated.

Furthermore, our study considered presence or absence of a diagnosis of any form of dementia, but did not collect information regarding behavioural or psychological symptoms, which have been shown to be more conducive to burden than cognitive deficits [26].

Finally, a recent systematic review has observed that, like many self-reported questionnaires on disability, the Katz Index of ADLs does not cover every essential domain of functioning, disability, and health [25]. Accordingly, the ADL scores used in this study may not reflect aspects of functioning which may bear on caregiver burden.

## Conclusions

In a real world setting, our study suggests that patient physical frailty may worsen caregiver burden, including associated psychological and physical morbidities. As such, it indicates that future observational and experimental studies addressing physical frailty may benefit from including caregiver burden as a dependent variable. It also provides further support for the observation that “the inclusion of carers in trials targeting frailty may assist in the identification of at-risk carers and facilitate the provision of information and support that will assist them in their role.” [27] In addition to addressing some of the limitations discussed above, future studies might be expanded to include analysis of caregiver characteristics or consider support structures. For instance, other studies have shown that caregiver gender, competence, coping and personality traits, and health may affect burden, and that respite care may moderate caregiver burden [26, 28, 29].

## Abbrevations

ADL: Activities of daily living
FFS: Fried Frail Scale
ZBI: Zarit Burden Interview
